# Effects of the isoflavone genistein in early life stages of the Senegalese sole, *Solea senegalensis*: role of the Survivin and proliferation versus apoptosis pathways

**DOI:** 10.1186/s12917-018-1333-3

**Published:** 2018-01-17

**Authors:** Carmen Sarasquete, María Úbeda-Manzanaro, Juan B. Ortiz-Delgado

**Affiliations:** Instituto de Ciencias Marinas de Andalucía-ICMAN.CSIC-Campus Universitario Río San Pedro, Puerto Real, 11510 Cádiz, Spain

**Keywords:** Genistein, Metamorphosis, Senegalese sole, Transcripts; immunohistochemistry, Proliferation, Apoptosis, Survivin

## Abstract

**Background:**

Phytochemical flavonoids are widely distributed in the environment and are derived from many anthropogenic activities. The isoflavone genistein is a naturally occurring compound found in soya products that are habitual constituents of the aquafeeds. This isoflavone possesses oestrogenic biological activity and also apoptotic properties. The present study has been performed to determine the effects of the genistein in the early life stages of the flatfish Senegalese sole during the first month of larval life, and it is focused especially at the metamorphosis, analysing the expression transcript levels and the immunohistochemical protein patterns implicated in the cell proliferation and apoptosis pathways (proliferation cellular/PCNA, anti-apoptosis Survivin/BIRC-5, death receptors/Fas, and Caspases).

**Results:**

The isoflavone genistein induced some temporal disrupting effects in several pro-apoptotic signalling pathways (Fas, CASP-6*)* at both genistein doses (3 mg/L and 10 mg/L), with increased Fas transcripts and also decreasing CASP-6 mRNA expression levels during metamorphic and post-metamorphic stages of the Senegalese sole. On the other hand, the anti-apoptotic BIRC-5 expression levels were weakly down-regulated with both the highest and lowest doses, but all of these imbalances were stabilised to the baseline levels. In early life stages of the controls, the constitutive basal transcript levels were temporarily and differentially expressed, reaching the highest levels at the pre-metamorphosis phase, as especially in endotrophic larvae (i.e. BIRC-5 mRNA), as well as in the metamorphic (i.e. CASP-6 mRNA) and post-metamorphic stages (i.e. Fas mRNA). In general, through development, continuous and progressive increases in the protein patterns of cell proliferation-PCNA (e.g. mitotic nuclei), anti-apoptotic Survivin (e.g. haematopoietic system, brain, digestive system, gills) and CASP-2 and -6 (e.g. brain, gills, kidney, digestive system, vascular systems, among others) have been immunohistochemically detected. Besides, both the controls and genistein exposed larvae displayed parallel immunostaining protein patterns in the different organ-systems and tissues.

**Conclusions:**

The transcriptional imbalances observed in the studied genes (BIRC-5, CASP-6, Fas) were only temporarily induced, and apparently no changes in the immunohistochemical protein patterns were detected. Thus, the isoflavone genistein caused not harmful effects in the development and metamorphosis of the Senegalese sole exposed to chronic environmentally relevant concentrations (3 and 10 mg/L).

## Background

Numerous references about environmentally relevant concentrations of many xenobiotics acting as endocrine disrupting compounds (EDCs) or as selective endocrine oestrogen receptor modulators (SERMs) have been published for the last twenty years, including analyses of several phytochemicals (isoflavones, phytosterols, coumestans, lignans) in the environment, and studies about the effects of phytoestrogen exposure on fish species [1–9], among others. As a result of different anthropogenic activities, several phytoestrogenic isoflavones (genistein, daidzein, equol, etc.) are discharged in the environment. Currently, variable concentrations of isoflavones and other phytochemicals from nanomolar range to levels up to 0.25 mg/L have been reported in different worldwide ecosystems [5, 10–13]. On the other hand, different vegetal compounds are used to partially substitute fishmeal in the feedstuffs, including the soya bean meal, which have a high protein content, good amino acid profile, palatability to fishes, availability and low price [3, 14–16]. Nevertheless, most of plant-derived products (e.g. soya, corn, wheat, etc.) utilised in aquafeeds also contain oestrogenic and anti-nutritional factors (i.e. flavones, phytosterols, saponins, trypsin inhibitors, lectins, antigens, etc.), contributing to several disorders in different fish species [17, 18].

In fish, sensitivity, toxicity and harmful effects of many different oestrogenic compounds (e.g. xenoestrogens, phytochemicals, etc.) vary depending on the species, life stages and developmental patterns, as well as toxicant-type, concentration and routes of exposure, absorption, uptake, metabolism, and detoxification [8, 9, 18–21]. Nevertheless, as far as we know, there are very few studies on the effects of flavonoids in the larval fish development and, particularly, focusing on the metamorphosis process of flatfish species, like Senegalese sole [22]. The majority of phytoestrogens can induce both agonist and antagonist effects at oestrogenic and thyroidal levels, among other hormonal disruptions or endocrine modulations [3, 20]. Genistein (4′,5,7-trihydroxyisoflavone) and other phytoestrogens can bind to oestrogen receptors (ERs) and activate ER-dependent pathways, acting more like SERM than like typical oestrogen agonists; that is, depending on the profile of co-activator and co-repressors proteins present in the cell, it can act as ER agonist or ER antagonist. Furthermore, isoflavones have also shown oestrogen-independent effects, via apoptotic pathways [3, 4, 6, 7, 23, 24]. Additionally, it is very well known that protein tyrosine kinases -PTKs- (i.e. phosphorylation of proteins) participate in all proliferation processes (DNA-replication) and these kinases can act as intermediary and/or alternative non-genomic mechanisms related with the induction of apoptosis, that also is modulated (e.g. inhibition of PTKs) by flavonoids, such as the isoflavone genistein [3, 25, 26].

On the other hand, homeostasis of multicellular organisms is controlled not only by proliferation and cell differentiation but also by cell-death or apoptosis [27, 28]. The proliferation cell nuclear antigen (PCNA), also known as cyclin, is a 36-kDa multifunctional protein highly expressed in the nuclei during the G1 and S phases of the dividing cells, and this cyclin also plays an important role in DNA repair by interacting with the partner proteins [29–31]. Besides, it has been observed that the expression of PCNA -genes and proteins- correlates with cell proliferation and DNA replication [32]. In both normal and pathological tissues of fish species, immunohistochemistry of cell cycle related proteins (i.e. PCNA), which is specifically confined to the nuclei of dividing cells, is widely accepted as one of the most characteristic cellular and molecular marker of proliferation [33–37]. Unlike mammals, in fish species, the patterns of cell proliferation and rates of cell renovation display inter and intra-specific differences, depending on the normal developmental events, pathological conditions, nutritional or environmental stressors, among others [38–41].

Moreover, apoptosis or programmed cell death plays crucial roles in fundamental biological steps, such as embryonic morphogenesis, metamorphosis, and remodelling tissues, among other ontogenetic developmental events [42]. Apoptosis is regulated by different modulators, including genes (e.g. death receptors, Fas), proteins (e.g. Caspases, inhibition apoptosis proteins -IAPs) and even organelles (e.g. mitochondria, endoplasmic reticulum) [27, 43, 44]. At least in higher vertebrates, two sub-groups of caspases have been characterised in the apoptotic signalling pathway: the initiators (Caspase-2, −8, −9, −10) and the effectors or executioners (Caspase-3, −6, −7); whereas the inflammatory caspases (Caspase-1, −4, −5, −11, −12, −13) are included in other subfamily [43, 44]. Although the apoptotic machinery is well conserved between aquatic and terrestrial organisms, some differences exist in key components of the extrinsic and intrinsic pathways or apoptosis; for example, the Fas-associated death domain protein of fish, birds and frogs lacks the C-terminal extension present in mammals [42, 45, 46]. Among other tumour necrosis factors (TNFs), one of the most extensively studied death receptors is the stimulating apoptosis factor (Fas), also known as apoptosis antigen-1, or cluster of differentiation-95 [42–44]. The mechanisms underlying apoptosis induced by Fas and Fas ligand (FasL) interactions have been studied in a variety of pathological circumstances, in which the up-regulation of these factors seems to result in dependent-cell death [27, 42–44].

Besides, the Survivin (i.e. gen BIRC-5), which is a member of the inhibitor of apoptosis protein (IAP) family, is a fascinating little protein with dual roles in promoting cell proliferation and preventing apoptosis, and it is considered as a protein that interfaces life and death [28, 44, 47]. Inhibition of apoptosis is necessary to cell survival, and this is carried out by inhibiting the expression of pro-apoptotic factors (i.e. Caspases, death receptors), as well as promoting the expression of anti-apoptotic factors [44]. IAPs are characterised by a domain termed the baculoviral IAP repeat -BIR- and these proteins require both the presence of BIR domain and the ability to suppress apoptosis [48–51]. A high expression of Survivin in embryonic and early development may contribute to tissue homeostasis, development and differentiation, but the BIRC-5 gene then becoming quiescent in most normal adult tissues [42–44].

Many ontogenetic studies have been performed at physiological, molecular, biochemical, histological and cellular levels in the Senegalese sole development [52–54]. Nevertheless, in fishes, and particularly in flatfish species that suffer a complex ontogenetic transitional and thyroid-driven metamorphosis [22, 52–55], little research has focused on comparative molecular and cellular analysis of cell proliferation and/or apoptosis patterns [33–37]. However, both cell-life and cell-death pathways are crucial and complementary events necessary for controlling the homeostasis, development, differentiation, growth and survival [27, 28, 34, 42].

The main ontogenetic events of the Senegalese sole, enclosing several important morphological, histological, immunohistochemical characteristics, as well as biometric parameters described during the first month of larval life have been reported recently [22]. Accordingly, the classification into pre-metamorphic phases (P1-P9, from 2.4 mm to 4.4 mm), early, middle and later metamorphic stages (S1, S2, S3, from 4.5 mm to 9.9 mm) and the post-metamorphic stage (S4, from 10 mm to 12.mm, from 23 dph onwards) have been updated by these last authors. Additionally, many important ecological, biological and physiological ontogenetic particularities have been reported during the larval development of this flatfish species [22, 52–55], among others. Based on all of these previous studies and taking in consideration the oestrogenic and pro-apoptotic properties of the isoflavones (i.e. genistein), the aim of this work is to analyse the sub-lethal effects of the soya isoflavone genistein, in early life stages (ELS) of the flatfish Senegalese sole, *Solea senegalensis*, from the end of the pre-metamorphic phases (from from 9 dph, P9 on, low doses at 3 mg/L), and the middle of metamorphic phases (from 16 dph, high doses at 10 mg/L) to the post-metamorphic stages (from 23 dph onwards, S4). In this sense, the dose around 3 mg/L is closely to the lethal concentration (LC50) determined in the early life stages of other fish species, like zebrafish, whereas the maximum genistein solubility could be reached at around 10 mg/L [4, 6, 7]. In addition, the development of both the controls and exposed Senegalese sole ELS to chronic environmentally relevant concentrations of the genistein (3 mg/L and 10 mg/L) of have been recently summarised and updated [22]. We emphasised on the metamorphosis process, and focused on mitotic dividing-cells, and apoptosis signalling pathways, such as cell proliferation (i.e. PCNA), anti-apoptosis (Survivin/BIRC-5), death receptors or stimulating apoptosis factor receptor (Fas) and pro-apoptosis signals (Caspases-2, −6), which have been analysed at both the molecular transcriptional and the immunohistochemical protein levels.

## Methods

### Biological samples

Newly hatched Senegalese sole larvae were provided by Research Facilities of Aquaculture-IEO (Santander, Spain), and maintained in 16 L cylinder tubes from hatching to 30 days post-hatching (dph) at the Institute of Marine Sciences of Andalusia (ICMAN-CSIC). Our facilities, in agreement with the European Convention for the Protection of Animals used for Experimental and Scientific purposes, were approved for experimentation by the Ministry of Agriculture and Fisheries (REGA-ES110280000311) in accordance with current EU (Directive 2010/63) and Spanish legislation. The experimental procedure (project AGL2014–52906-R) was approved by the Spanish National Research Council (CSIC) Ethics Committee, and dependent Spanish Competent Authority of Junta de Andalucía (n° 09–7–15-278, RD53/2013).

Larvae were maintained at 18 ± 1 °C, water change every 24 h, and a 12 L:12D photoperiod (light intensity of 600–800 lx). Larvae were fed daily with marine rotifers (*Brachionus sp*) from 3 to 9 dph, with artemia nauplii from 7 to 20 dph, and with enriched artemia metanauplii form 21 dph until the end of the experiment, such as it was also recently described [22].

Genistein (C_15_H_10_O_5_, LC Laboratories, MA, USA) was dissolved in ethanol to make up 20 mM solution stock, and then was kept in darkness at 4 °C. Tubes were randomly assigned in duplicate as control groups (with and without the carrier, ethanol) and as two isoflavone-treated groups of 3 mg/L (performed on fish of 9 dph to 26 dph) and 10 mg/L (from 16 dph to 29 dph). All treatment tubes were completely renewed daily with freshly prepared stock solution. No difference in mortality was observed between controls and genistein-exposed groups.

After anaesthesia with 500 ppm phenoxyethanol, samples of pelagic larvae, larvae undergoing metamorphosis and post metamorphic individuals were collected as pools of larvae (*n* = 3) in triplicate for molecular analysis (into RNAlater^®^, Sigma-Aldrich, incubated for 24 h at 4 °C and stored at −80 °C until RNA extraction), and for immunohistochemistry and histological procedures (samples were fixed with 4% paraformaldehyde in diethylpyrocarbonate treated phosphate-buffered saline (PBS) 1X overnight at 4 °C and stored in methanol at −20 °C after washing 3 times for 1 h with PBS 1X), following our standardised technical protocols which were adapted to this specific research [37, 56].

### Nucleic acids extraction and quantification of mRNA expression levels

Total RNA was isolated from pooled larvae using RNeasy^®^ Micro kit or RNeasy^®^ Mini kit (Qiagen) according to the manufacturer’s protocol. Genomic DNA was removed via on-column DNase digestion at 37 °C for 30 min using DNase (RNase-free included in the kit). Total RNA quality was verified on a Bioanalyzer 2100 (Agilent Technologies) and its concentration was assessed by spectrophotometry (A260 nm/A280 nm ratio > 1.9).

For cDNA synthesis, 500 ng of total RNA was used for reverse transcription using a qScript™ cDNA Synthesis kit (BioRad) according to the manufacturer’s protocol. Real-time analysis was performed on a Mastercycler^®^ ep gradient S Realplex^2^. Each reaction was carried out in triplicate mixture containing 300 nM each of specific primer pair (Table 1), 4 μL of a 1/10 dilution of cDNA (≈10 ng), and 5 μL iTaq™ Universal SYBR^®^ Green SuperMix (BioRad) in a final volume of 10 μL. The qPCR profile was as follows: 95 °C for 2 min, 40 cycles at 95 °C for 15 s, 56 or 60 °C for 15 s (see Table 1), and 60 °C for 15 s. All primers gave single distinctive melting peaks, demonstrating the absence of primer-dimer artefacts. To confirm the correct amplification, the obtained amplicons were cloned and sequenced (pGem-T Easy Vector System, Promega). Relative gene quantification was performed using the method of Pfaffl [57], and the results were normalised to elongation factor 1-alpha and 18S rRNA, with mRNA from the 1 dph larvae as calibrator. Negative qPCR controls using double-distilled water and RNA instead of cDNA were included in the assays for each primer pair.

**Table 1:** Primer sequences, amplicon length (AE), annealing temperature (T), efficiency (E) and gene reference

Target	Sequence (5’→3′)	AE (bp)	T (°C)	E^a^	Gene reference
BIRC-5	TGATCCAGAAAAGGAGCACA (F); TGGTGTCGTTGCAGGATTTA (R)	149	56	2.0799	solea_v4.1_unigene59754*
Caspase-6	CAGGACATCACAGCCATGTT (F); TCACTGTCCACAGCATCACA (R)	134	60	2.0205	solea_v4.1_unigene3643*
Fas	AGCCACTGCTCTGTGAAACC (F); CTAGGTTTGCGTTGGGATGT (R)	130	60	2.0403	solea_v4.1_unigene23355*

^a^100% efficiency is E = 2 and corresponds to a slope of −3.32. to accept the standar curve the R^2^ value must be >0.99

*Database Solea BD. http://www.juntadeandalucia.es/agriculturaypesca/ifapa/soleadb_ifapa/

### Statistical analysis

Temporal differences of the gene expression of control larvae within each developmental stage were tested using one-way analysis of variance (ANOVA) performed after logarithmic base 10 transformation to fulfil the requirements for parametric ANOVA. Normality was checked using the Shapiro-Wilk’s test, and the homogeneity of variances with the Levene’s test. Tukey’s post hoc test was used to identify significantly different groups. A t-Student test was performed to identify significant differences in gene expression between each treatment and its control. Differences were considered statistically significant at *p* < 0.05. Statistical analyses of data from qPCR were performed using SPSS 23.0.0.0 software (IBM).

The assessment for immunohistochemical results was performed independently by three observers, which analysed the same histological samples. Similar immunostaining results were tested and confirmed analysing three samples per observer. From each sample, two histological slides of control specimens (plus solvent) and of each experimental assay (high and low concentrations) were analysed, from 10 dph onwards.

### Histology and Immunohistochemical detection

The samples were embedded in paraffin to obtain 6 μm histological serial sections of whole larvae. The Haematoxylin-Eosin and Haematoxylin-VOF techniques [58] were used to verify the histomorphological characterisation of larval development.

The immunohistochemical approach was carried using the commercial primary antibodies caspase-2 (3027–100, BioVision, polyclonal rabbit anti-mouse caspase-2), caspase-6 (AB10512, EMD Millipore, polyclonal rabbit anti-rat caspase-6), BIRC-5/Survivin (#2808, Cell Signalling, monoclonal rabbit anti-human survivin), and PCNA (sc-56, Santa Cruz Biotechnology, monoclonal mouse anti-rat PCNA), according to Ortiz-Delgado et al. [37]. Assayed dilutions of antibodies were 1:250. As commercial primary antibodies against caspase-2, caspase-6, BIRC-5 and PCNA of fish are unavailable, the cross-reactivity and specificity of these antibodies development against mammalian antigens were previously tested: the percentages of the amino acid sequences similarity were calculated using Basic Local Alignment Search Tool BLAST^®^ (https://blast.ncbi.nlm.nih.gov/Blast.cgi) and epitope similarities were compared, proving to be conserved for this and other fish species [34, 37], and were also validated based on their assumed pattern of reactivity within tissue sections of controls. At the same time, to confirm the specificity of the primary antibodies and the immunostaining results, positive and negative controls have also been tested; for instance, replacing primary antibody with pre-immune serum or bovine serum albumin and incubating with secondary antibody only (BA-2000 Vector, biotinylated horse anti-mouse IgG, at 1:50 for PCNA, and BA-1000 Vector biotinylated goat anti-rabbit IgG, at 1:50 for BIRC-5, Caspase-2 and Caspase-6); as well as by omission of secondary antibodies.

## Results

**Fig. 1:**
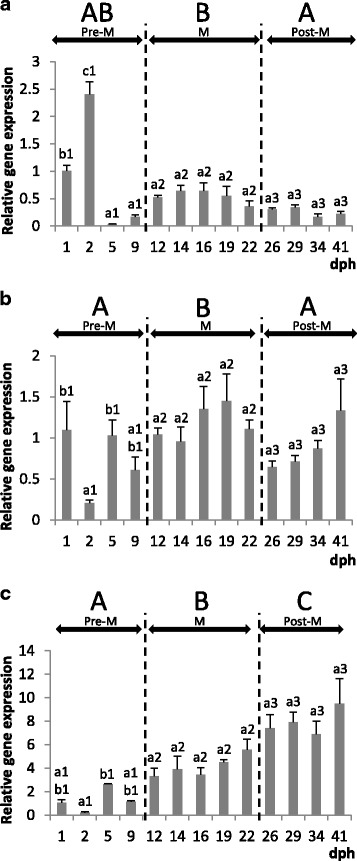
Relative expression levels of BIRC-5 (**a**), CASP-6 (**b**) and Fas (**c**) genes during larval development in Senegalese sole (mean ± SE, *n* = 3). Statistically significant differences within each developmental stage are separately indicated by lower case letters (a, b or c), while the adjacent number refers to the stage: 1 (Pre-M: pre-metamorphosis), 2 (M: metamorphosis), and 3 (Post-M: post-metamorphosis). Significant differences between each development stage groups are indicated by upper case letters (A, B or C). Differences were detected by ANOVA, Tukey post hoc test, *p* < 0.05

During development of the Senegalese sole, *S. senegalensis*, the expression pattern of BIRC-5 mRNA (anti-apoptotic Survivin, Fig. 1a) exhibited statistically significant differences into baseline profiles when comparing the metamorphic and post-metamorphic stages, whereas the pre-metamorphic stage presents very variable levels, reaching the highest expression levels in the lecitrotrophic larvae, at 1–2 dph. From this endotrophic phase on, a noticeable fall in the BIRC-5 mRNA levels was detected, just at the onset of the first exogenous feeding phase (at around 5 dph), and through the pre-metamorphosis phases. Furthermore, coinciding with the onset of the metamorphosis (S1, at around 12 dph onwards) a slight increasing trend has been detected, and the constitutive BIRC-5 baseline transcript levels were maintained at similarly elevated expression levels during all the metamorphic stages (from S1 to S3) in comparison with those post-metamorphosed specimens (S4), from 26 dph onwards. On the other hand, during the first month of larval life, the highest constitutive Caspase-6 expression levels (Fig. 1b) have been registered in metamorphic stages (S1-S3, from 10 to 12 dph until 22 dph), showing also similar and moderately elevated expression levels in pre-metamorphic phases (P1-P9) as well as in post-metamorphosed larvae (from 26dph onwards). In addition**,** the lowest constitutive Fas transcript levels were registered at the lecitotrophic phase (at around 2 dph), with progressive and statistically significant increases from 5 dph on, and particularly from the onset of the metamorphosis (at around 12 dph) until the end of the experimental period. Therefore, the highest Fas transcript expression levels were registered in post-metamorphic specimens (from 23 dph onwards), in comparison with pre and metamorphosing ELS of the Senegalese sole (Fig. 1c).

**Fig. 2:**
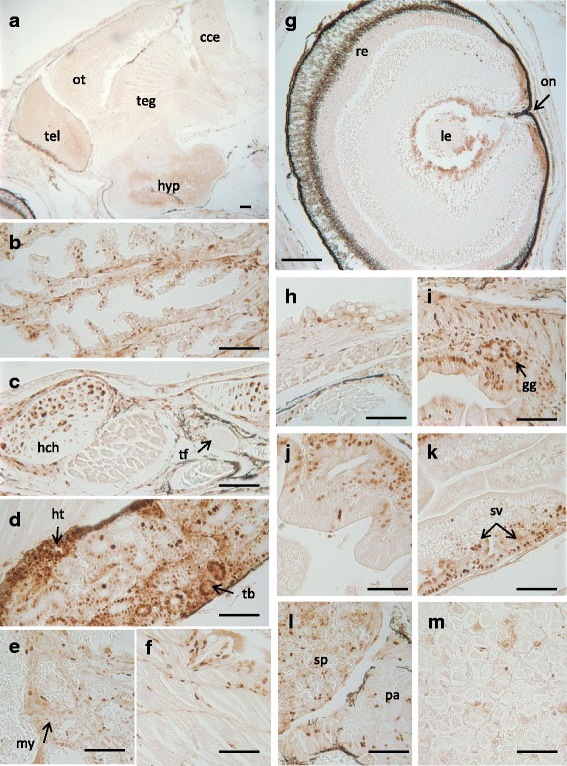
Immunohistochemical localisation of PCNA in control 29 dph larvae of Senegalese sole. Brain (**a**), gills (**b**), connective tissue of pharyngeal and thyroid gland (**c**), kidney (**d**), ventricle (**e**), musculoskeletal tissue (**f**), eye (**g**), oesophagus (**h**), stomach (**i**), anterior intestine (**j**), posterior intestine (**k**), pancreas and spleen (**l**), and liver (**m**). Scale bars represent 50 μm. cce: corpus of the cerebellum; gg: gastric glands; ht.: haematopoietic tissue; hch: hypertrophic chondrocytes; hyp: hypothalamus; le: lens; my: myocardium; on: optic nerve; ot: optic tectum; re: retina; sv: supranuclear vesicles; tb: tubules; teg: tegmentum; tel: telencephalon; tf: thyroid follicle

**Fig. 3:**
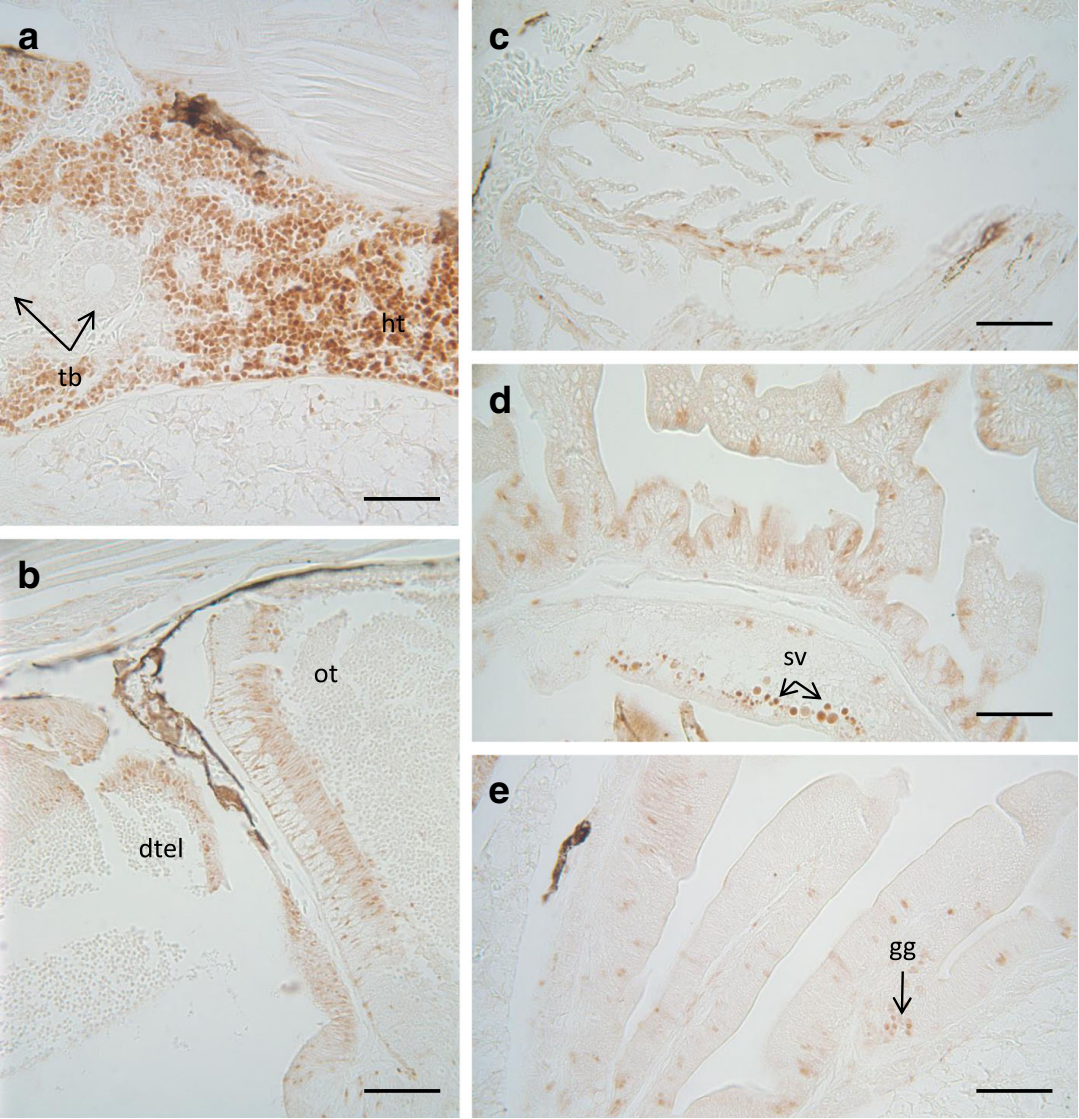
Immunohistochemical localisation of BIRC-5 in control 29 dph larvae of Senegalese sole. Kidney (**a**), brain (**b**), gills (**c**), intestine (**d**), kidney (**e**). Scale bars represent 50 μm. dtel: dorsal telencephalon; gg: gastric glands; ht: haematopoietic tissue; ot: optic tectum; sv: supranuclear vesicles; tb: tubules

**Fig. 4:**
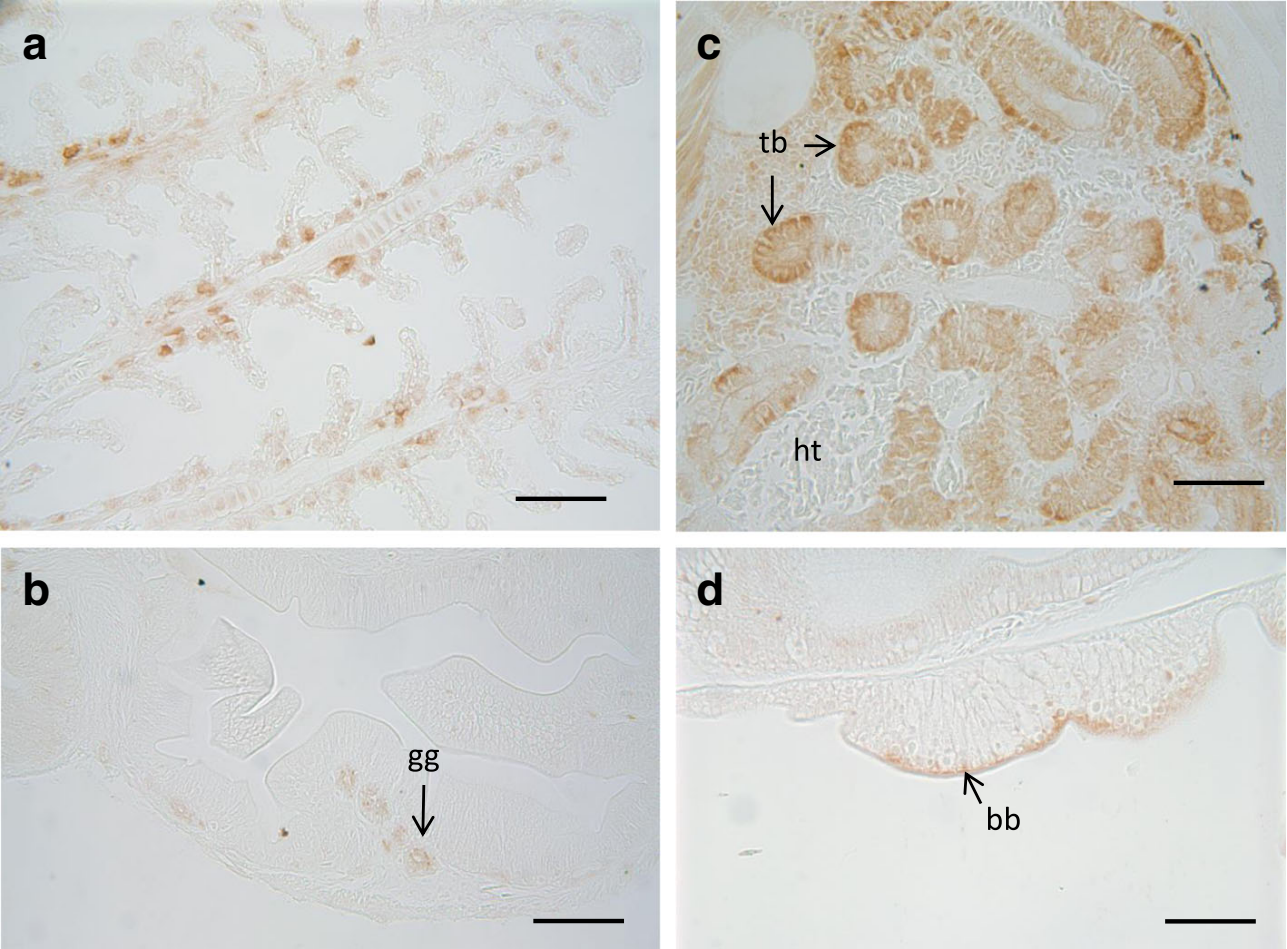
Immunohistochemical localisation of CASP-6 in control 29 dph larvae of Senegalese sole. Gills (**a**), stomach (**b**), kidney (**c**), intestine (**d**). Scale bars represent 50 μm. bb: brush border; gg: gastric glands; ht: haematopoietic tissue; tb: tubules

**Fig. 5:**
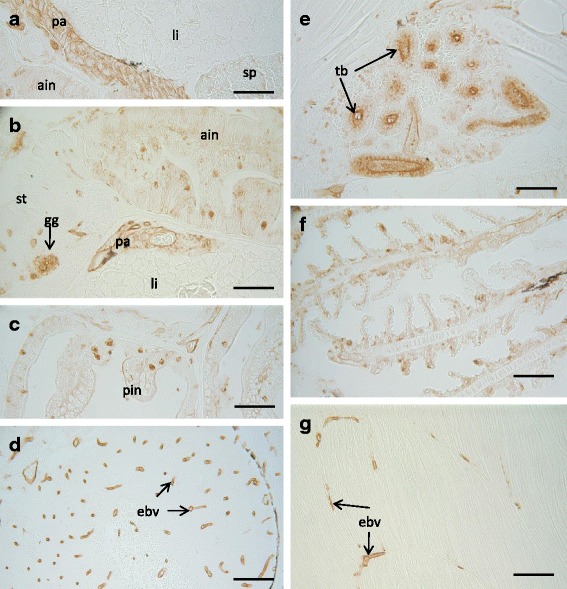
Immunohistochemical localisation of CASP-2 in control 29 dph larvae of Senegalese sole. Digestive system (**a**, **b**, and **c**), brain (**d**), brain (**e**), gills (**f**), muscle (**g**). Scale bars represent 50 μm. ain: anterior intestine; ebv: endothelium of blood vessels; gg: gastric glands; li: liver; pa: pancreas; pin: posterior intestine; sp: spleen; st: stomach; tb: tubules

Additionally, similar or parallel developmental patterns for PCNA, Survivin and CASP-2 and -6 were evidenced in Senegalese sole controls and exposed to both genistein doses, since there is no clear evidence of variations of the immunohistochemical staining patterns. Nevertheless, the strongest immunostaining staining patterns for CASP-6 and CASP-2 were registered at the metamorphic stages. The PCNA antibody displayed positive affinity in the majority of the organ-systems and cell-tissues, such as is represented in the Fig. 2, with specific and strong immunostained nuclei in certain brain areas (telencephalon, optic tectum, hypothalamus), eye (retina, optic nerve), gills (epithelial respiratory cells), hypertrophic chondrocytes, haematopoietic cell-tissue and some renal tubules. Furthermore, positive PCNA immunoreactivities were also detected in the digestive system and associated organs (gastric glands, intestinal enterocytes, supranuclear vesicles, exocrine pancreas, hepatocytes), as well as muscle-skeletal tissues and ventricle (Fig. 2). In addition, the immumohistochemical pattern for the Survivin (Fig. 3) displayed a specific and strong immunostaining, particularly in haematopoietic tissue of the kidney, as well as in specific brain areas (optic tectum and dorsal telencephalon). Besides, some Survivin immunoreactivities were moderately displayed in the respiratory epithelial cells, and in the digestive system (enterocytes and supranuclear vesicles of the posterior intestinal region, and, also some isolated stomach-gastric glands). On the other hand, the CASP-6 immunoreactivity was mainly displayed in gills, renal tubules, gastric glands and intestinal brush border (Fig. 4). Finally, the immunohistochemical pattern for CASP-2 was in general ubiquitously distributed in the majority of the organ-systems and cell-tissues. Thus, from moderated to strong immunostaining was evidenced in the exocrine pancreas, intestinal mucous cells, gastric glands, endothelia of the vascular system of the brain and skeletal muscle; as well as in epithelia of the renal tubules (Fig. 5).

**Fig. 6:**
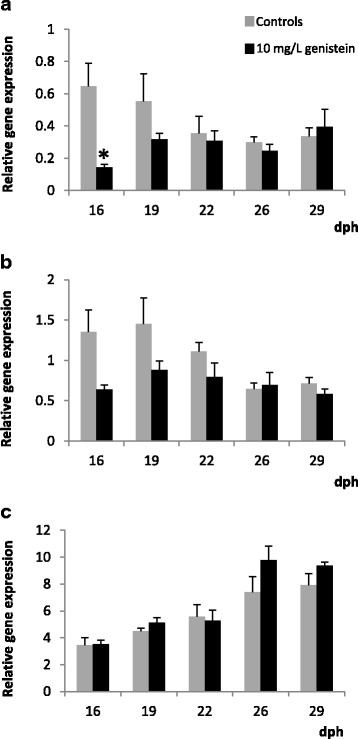
Relative expression levels of BIRC-5 (**a**), CASP-6 (**b**) and Fas (**c**) genes in the untreated control (grey) and 10 mg/L genistein-treated (black) groups (mean ± SE, *n* = 3). Values with an asterisk are significantly different (t-Student, *p* < 0.05) from the corresponding value for the control group

**Fig. 7:**
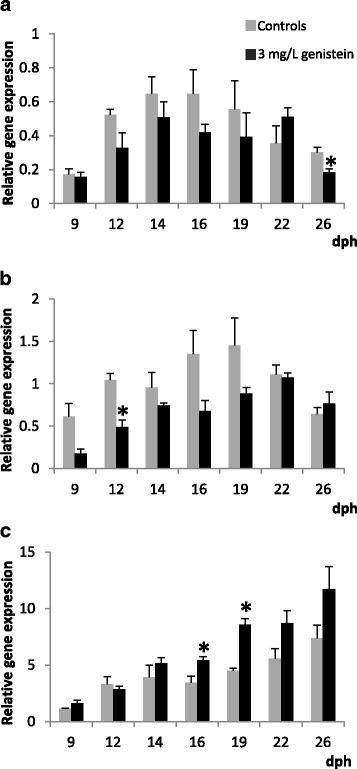
Relative expression levels of BIRC-5 (**a**), CASP-6 (**b**) and Fas (**c**) genes in the untreated control (grey) and 3 mg/L genistein-treated (black) groups (mean ± SE, *n* = 3). Values with an asterisk are significantly different (t-Student, *p* < 0.05) from the corresponding value for the control group

In the developing larvae exposed to 10 mg /L of genistein a statistically significant down-regulation of the anti-apoptotic BIRC-5 transcript levels (Fig. 6a) was registered at the middle metamorphic stage (S2, at 16 dph), as well as in post-larvae (S4, at around 26 dph) with the lowest doses (3 mg/L, Fig. 7a). Thus, a weak decreasing trend of anti-apoptotic signal BIRC-5 by effect of the genistein it has been detected, but only temporarily since BIRC-5 transcripts were restored at similar baseline expression levels than the controls. Interestingly, the expression patterns of both signals mediated in the apoptosis cascade pathways, CASP-6 and Fas, showed different responses by effects of the exposure to both genistein concentrations (Figs. 6b, c, 7b, c). In addition, some considerable variations in the regulation of pro-apoptotic factors have been induced by genistein. In general, both genistein doses seem to provoke that CASP-6 mRNA levels decrease apparently (no significantly), being only statistically significant at around 12 dph with the lowest genistein doses (3 mg/L). Nevertheless, the CASP-6 transcript levels are progressively more similar to the controls at the end of the metamorphosis and the post-metamorphic stages. On the contrary, the expression levels of the death receptor (Fas) transcripts increased (at around 16 and 19 dph) at the lowest genistein doses. Nevertheless, with the highest concentration of genistein tested, not statistically significant variations were registered.

Finally, Senegalese sole ELS from both controls and exposed to both genistein doses showed a normal organogenesis, larval development, metamorphosis, and growth (i.e sizes and length). In all experimental designs, just as in controls, around 70–80% of the specimens finished the metamorphosis process (from 10 to 12 dph until 20–22 dph), and they adapted to benthic life. In both groups, controls and exposed to genistein mortality rates and pigmentation anomalies were about 20–30%.

## Discussion

Unlike mammals, several fish species grow continuously throughout their life with little senescence, so all their cells would need a high proliferation capacity, and thereby high telomerase activities should be active in all fish cells [30, 44, 59]. It is well known that homeostasis of multicellular organisms is controlled not only by proliferation and differentiation of cells but also by cell death or apoptosis pathways [27, 44]. In addition, there are fascinating anti-apoptotic proteins (IAPs), such as Survivin with dual roles in promoting cell proliferation and preventing apoptosis [27, 28, 30, 42]. On the other hand, phytochemical flavonoids, such as the isoflavone genistein, act more likely as selective oestrogen receptor modulators (SERMs) because depending on the profile of co-activator or co-repressor proteins in the cell, the genistein can act in an agonist or antagonist way [3, 16]. Such as it was recently reported in the metamorphosis of the Senegalese sole, the genistein up-regulated the levels of ERβ mRNA at high concentrations (10 mg/L) but they were down-regulated at low concentration (3 mg/L). Nevertheless, these oestrogenic transcriptional disruptions were quickly restored to the basal levels, suggesting that genistein may act as SERM in the metamorphosis of this flatfish [22]. In addition, genistein can also activate oestrogen-independent-pathways, including pro-apoptosis signalling cascades, such as it has been reported in several vertebrates including fish species [3, 4, 14–16]. Accordingly, some flavonoids can inhibit pathways important for cell proliferation and growth, through PTK pathways, and this potential inhibitory capacity of the flavonoids may affect to multiple organ systems and cell-tissues [15, 16].

In the development of the Senegalese sole controls, all of the proliferation and apoptosis signalling genes analysed at the transcriptional level, as well as the protein patterns assessed by immunohistochemistry, were differentially expressed from early larval phases, as especially in the endotrophic larvae onwards. In general, continuous and parallel immunohistochemical staining patterns of cell proliferation (PCNA) and for anti-apoptotic Survivin protein patterns have been evidenced from pre-metamorphosis until post-metamorphosis. Besides, the highest immunoreactivities (i.e. PCNA and Survivin) in the different organ-systems and cell-tissues have been noted at the pre-metamorphic phases and metamorphic stages. These early life stages showed the highest expression levels of BIRC-5 and strong Survivin immunostaining patterns, and the lowest Fas transcript levels have been registered in these newly larvae. In addition, CASP-6 was also expressed at moderately high levels, in both the pre- and post-metamorphic phases, reaching the highest expression levels for CASP-6 in the metamorphic stages, while the highest Fas transcript levels are registered in post-metamorphosed larvae. All of these anti and pro-apoptotic signals which are expressed from ELS of the Senegalese sole, may derive from maternal sources, such as it was reported for other genes and dependent-proteins (oestrogen receptors, thyroid signals, among others) [22, 42, 46]. A precocious expression of all these genes may be necessary for homeostasis, proliferation and apoptotic processes during larval development and particularly at the metamorphosis of flatfishes. Indeed, both pro-apoptotic signals (Fas, CASP-6), which are expressed very early in the development of the Senegalese sole, should be necessary for the execution of the programme of cell death or apoptosis, serving as a fail-safe mechanism in early development to eliminate physiological damaged embryos and larval cells [42, 46]. Similarly, the patterns of active cell proliferation (stained mitotic nuclei assessed by PCNA immunohistochemistry) have been evidenced from pre-metamorphosis and during metamorphic and post-metamorphic stages, and it in parallel with development, differentiation, maturation of the organs-systems and cell tissues, such as it has been previously reported in this and other fish species [34–37]. In addition, the high expression of BIRC-5 (anti-apoptotic Survivin) in early life stages of the Senegalese sole may play a crucial role, and it may contribute to tissue homeostasis, as well as for promoting proliferation and preventing apoptosis [42–44], what is of particular physiological importance in these critical and sensitive early larval phases, and especially of flatfishes [21, 22, 24, 34, 52–55], among others.

Proliferating cell nuclear antigen (PCNA) is expressed in sites of rapid cellular proliferation, and also plays a role in DNA repair and apoptosis by interacting with different partner proteins [30, 31, 60, 61]. In the current study, gene expression levels of PCNA were not analysed, but it is widely accepted that both PCNA -mRNA transcripts and proteins- correlate with cell proliferation and DNA replication [21, 32–37]. During larval development of the Senegalese sole, in general progressive and strong PCNA immunostaining patterns have been evidenced from pre-metamorphic phases, and during the active metamorphic stages. In addition, because of its crucial proliferation function, PCNA immunostaining displayed developmental patterns in parallel with the progressive differentiation and maturation of the different organ-systems and cell-tissues (i.e. brain development, migration of eye, digestive and thyroid ontogeny, skeletal remodelling, among others), which are the most characteristic ontogenetic developmental events of flatfish species, such as Senegalese sole [22, 53, 62, 63]. In fact, the proliferation zones described in the brain (preoptic region, dorsal thalamus, posterior tubercle, hypothalamus, etc.) at the pre-metamorphic phase are conserved until later developmental stages, long after metamorphosis of the Senegalese sole, such as it has been previously pointed out [34]. Furthermore, PCNA is widely detected in haematopoietic stem cells and neuro-retinal cells, as well as in the digestive glands, gills, operculum, bone, among other cell tissues, during the embryogenesis and larval development, such as it has been detected in several different larvae, juvenile and adult fish species [35, 37–39, 60, 61, 63]. PCNA positive cells are confined within the haematopoietic compartment, and in close proximity to the endothelial cells, suggesting possible cross-talk between haematopoietic, endothelial and renal tubular cells, such as it was reported in other fish species [35, 61]. In several teleost species feed with vegetal compounds (i.e. soya beans), or exposed to contaminants, increased PCNA-positivity in intestinal mucosa have been related with a higher rate of cell renewal [38, 41, 64]. Nevertheless, in the present study, no discernible changes in the PCNA, Survivin and Caspases −6 and −2 immunohistochemical staining patterns have been detected, when comparing both the controls and ELS of the Senegalese sole exposed to genistein (low and high doses).

Survivin is the smallest member of the inhibitor of apoptosis protein (IAP) family which takes part in inhibition of apoptosis and regulation of cell cycle [28, 51]. In the present study, in progressive and parallel concordance with those previously described larval phases and metamorphic stages of the Senegalese sole development [22], the haematopoietic interstitial tissue of the head-kidney was especially and strongly Survivin-immunoreactive, as well as some specific brain areas (i.e. optic tectum, dorsal telencephalon). Moreover, a moderate Survivin immunostaining pattern was evidenced in gills, digestive system (e.g. gastric glands, supranuclear vesicles). In vertebrates, it is well known that several hormones, i.e. 17 -β oestradiol, with structural similarity to genistein, regulate the Survivin transcript expression levels and also modulate the cell proliferation phases [65]. Survivin shows an important role in embryonic and totipotent stem cell function, as well as, in the regulation of physiology of haematopoietic, neuronal or intestinal stem cells in adult organisms [44, 47–49, 51]. In addition, Survivin may play a role in maintaining gastric mucosal integrity and regulating proliferation and rates of cell renewal in the gastric mucosa of vertebrates, and most particularly in fish species [41]. Survivin has been mainly detected in the cytosol in where is believe that act as apoptotic suppressor, although there are different subcellular pools of Suvivin in nucleus and mitochondrion [51]. The mitochondrial Survivin is discharged in cytosol in response to apoptotic stimuli to suppress caspase activation [66], while nuclear Survivin is postulated to regulate cell division, corroborating the known dual role for Survivin, as regulator of both mitosis and apoptosis [28, 51].

On the other hand, in fish species, both the extrinsic and intrinsic pathways are active during development, although for instance zebrafish development proceeds normally without extrinsic pathways, suggesting that intrinsic pathway is the main mediator of embryological cell death during normal development [42]. In other species, e.g. Atlantic salmon, the effector Caspases (i.e. Caspase- 6) are widely expressed during embryogenesis, in several different organs system and cell-tissues (eye, somites, intestine, pronephros, pharyngeal arch, otolitic vesicle and brain, among others), such as it was revised by Takle and Andersen [42]. In the Senegalese sole development, CASP-6 showed specific and strong immunoreactivity mainly in gills, renal tubules, gastric glands and intestinal brush border. Interestingly, the immunohistochemical pattern of CASP-2 was in general ubiquitously distributed, showing from moderated to strong immunostaining in the majority of the organ-systems and cell-tissues, such as exocrine pancreas, intestinal mucous cells, gastric glands, endothelia of the vascular system of the brain, and skeletal muscle; as well as in epithelia of the renal tubules, among other tissues. The initiator caspase-2 and executioners caspases −6 and −3, among other pro-apoptotic factors, can be considered as adequate pro-apoptotic signalling markers [37, 42–44, 46, 67].

In different fish species, phytochemical flavonoids (i.e. isoflavone genistein), as typical phytoestrogens and apoptotic compounds, induced serious disturbances in several hormonal signalling pathways, provoking noticeable disorders during embryogenesis, larval development, growth and sex determination and differentiation [2–4, 6, 7, 23, 24], among others. Nevertheless, thyroidal and oestrogenic effects of genistein seem ambiguous, and both agonist and antagonist effects have been pointed in several vertebrates [6, 7, 67–70]. Such as it was recently reported during the larval development of the Senegalese sole, the potential disrupting effect at the oestrogenic (i.e. ERβ up-or down-regulation) and thyroid levels was only temporarily altered by the genistein, since weak increases or decreases of ERβ mRNA and several thyroid signalling transcripts were quickly restored to the baseline levels [22]. However, in zebrafish among other species, genistein binds to three ER types (ERα, ERβA, ERβB), corroborating that the phytoestrogen isoflavone can activate the oestrogen pathway through a direct binding to the ERs [4, 6, 7]. Besides, genistein also induced apoptosis by independent-oestrogenic pathways, possibly via PTKs, among other signals in vertebrates including several fish species [4, 6, 7, 23, 25, 26], among others. Nevertheless, in the current study, a specific apoptotic effect induced by the isoflavone genistein has not been established, at least by analysing some known markers integrated in the proliferation and apoptosis cascade pathways (PCNA, Survivin, BIRC-5, Caspases, Fas), which are known markers of both processes at both molecular and cellular levels [28, 42, 44]. During development of the Senegalese sole, BIRC-5 transcript levels were only weakly down-regulated (at 16 dph, and 26 dph) by effect of the isoflavone genistein (high and low doses), but these decreased BIRC-5 mRNA transcript levels were restored quickly to baseline expression levels similar to the controls. Moreover, coinciding with slight decreases of BIRC-5 transcript levels induced by the isoflavone (high and low doses), apparently also minor CASP-6 (apoptotic effector) mRNA transcript levels could be noted, mainly at around 12 dph (low genistein doses), showing temporal decreasing levels (no statistically significant differences) during all the metamorphic stages. In addition, the baseline transcript levels of the apoptosis death receptor (i.e. Fas) were up-regulated at the metamorphic stages with the lowest genistein doses (3 mg/L), but these responses were stabilised at the end of metamorphic stages (at around 22 dph on), and coincide also with significant apparent decreasing levels of CASP-6 mRNA (low genistein doses) at around 12 dph on. Accordingly, in general the proliferation and pro and anti-apoptotic signalling pathways appeared be stabilised at the end of the experimental assays, during the first month of larval life of Senegalese sole ELS exposed to the isoflavone genistein.

Finally, majority of Senegalese sole larvae (controls and exposed to genistein) showed a normal organogenesis, development, metamorphosis and growth patterns, similarly to data from many previous findings reported on this flatfish species [22, 52–55, 63, 71], and it is also in concordance with symptoms of healthy larvae and early juveniles. Additionally, the high rates of larval and juvenile survival, and similarly biometric parameters recorded also in the present study could be compatible, at least apparently, with no harmful effects induced by the exposure to the isoflavone genistein (3 mg/L and 10 mg/L), such as it has been previously suggested in this flatfish species [22]. As a first consideration and consequently, complementary and additional molecular, biochemical and cellular markers of apoptosis via intrinsic and extrinsic pathways, as well as the basal constitutive developmental patterns of cyclin or PCNA mRNA transcript levels, among other proliferation and apoptosis signalling pathways, could also be analysed.

## Conclusion

As a general conclusion from the current study where several proliferation and apoptotic (pro and anti) signalling pathways have been studied and compared in the newly hatched larvae and during the first month of larval life, is that no clear evidence of induction of apoptosis by the effect of the genistein, when using two known markers of apoptosis (death receptor Fas, and Caspase pathways) at the transcriptional level. Interestingly, at the protein level, no appreciable variation of PCNA, BIRC-5 and CASP-2 and -6 were evidenced in fish exposed to the genistein, suggesting an adequate process and balance of cellular proliferation versus apoptosis pathways, at both the transcriptional and protein levels, in both controls and genistein-exposed Senegalese sole ELS. Additionally, all of the Senegalese sole ELS analysed and compared, from both the controls and exposed to the genistein (low and high doses), showed a normal organogenesis pattern, optimal larval development, metamorphosis, and growth, since all specimens (80%) survived and they adapted correctly to benthic life during the first month of life.

## Abbreviations

EDC: Endocrine disrupting compound
ELS: Early life stages
ER: Oestrogen receptor
IAP: Inhibitor of apoptosis protein
PTK: Protein tyrosine kinase
SERM: Selective oestrogen receptor modulator
TNF: Tumour necrosis factor

## References

[1] Kiparissis Y, Balch GC, Metcalfe TL, Melcalfe C. Effects of the Isoflavones Genistein and Equol on the gonadal development of Japanese Medaka (*Oryzias latipes*). Environ Health Perspect. 2003;111:1158–63. 10.1289/ehp.5928 PMC1241568 12842767

[2] Ng Y, Hanson S, Malison JA, Wentworth B, Barry TP (2006). Genistein and other isoflavones found in soybeans inhibit estrogen metabolism in salmonid fish. Aquaculture.

[3] Patisaul HB, Jefferson W (2010). The pros and cons of phytoestrogens. Front Neuroendocrinol.

[4] Sassi-Messai S, Gibert Y, Bernard L, Nishio SI, Ferri-Lagneau KF, Molina J (2009). The phytoestrogen genistein affects zebrafish development through two different pathways. PlosOne.

[5] Rocha MJ, Cruzeiro C, Rocha E (2013). Quantification of 17 endocrine disruptor compounds and their spatial and seasonal distribution in the Iberian Ave River and its coastline. Toxicol Environ Chem.

[6] Schiller V, Wichmann A, Kriehuber R, Muth-Köhne E, Giesy JP, Hecker M (2013). Studying the effects of genistein on gene expression of fish embryos as an alternative testing approach for endocrine disruption. Comp Biochem Physiol C Toxicol Pharmacol.

[7] Schiller V, Wichmann A, Kriehuber R, Chafers C, Fisher R, Fenske M (2013). Transcriptome alterations in zebrafish embryos after exposure to environmental estrogens and anti-androgens can reveal endocrine disruption. Reprod Toxicol.

[8] Brown SB, Adams BA, Cyr DJ, Eales JG (2004). Contaminant effects on the teleost fish thyroid. Environm Toxicol Chem.

[9] Brown AC, Stevenson LM, Leonard HM, Nieves-Puigdoller K, Clotfelter ED (2014). Phytoestrogens β-sitosterol and genistein have limited effects on reproductive endpoints in a female fish, *Betta splendens*. Biomed Res Int.

[10] Kiparissis Y, Hughes R, Metcalfe C, Ternes T (2001). Identification of the isoflavonoid genistein in breached kraft mill effluent. Environ Sci Technol.

[11] Rearick DC, Fleischhaker NT, Kelly MM, Arnold WA, Novak PJ (2014). Phytoestrogens in the environment, I: ocurrence and exposure effects on fathead minnows. Environ Toxicol Chem.

[12] Ribeiro AR, Maia A, Santos M, Tiritan ME, Ribeiro CMR (2016). Occurrence of natural contaminants of emerging concern in the Douro River estuary, Portugal. Environ Contam. Toxicology.

[13] Spengler P, Korner W, Metzger JW (2001). Substances with estrogenic activity in effluents of sewage treatment plants in southwester Germany. 1. Chemical analysis. Environ Toxicol Chem.

[14] Setchell KD, Cassidy A (1999). Dietary isoflavones: biological effects and relevance to human health. J Nut.

[15] Wang Q, Ge X, Tian X, Zhang Y, Zhang JE, Zhang P (2013). Soy isoflavone: the multipurpose phytochemical (review). Biomed Rep.

[16] Hussain H, Green IR. A patent review of the therapeutic potential of isoflavones (2012-2016). Expert Opin Ther Pat. 2017;13:1–12. 10.1080/13543776.2017.1339791 28586284

[17] Francis G, Makkar HP, Becker K (2001). Antinutritional factors present in plant-derived alternate fish feed ingredients and their effects in fish. Aquaculture.

[18] Gatlin DM, Barrows FT, Brown P, Dabrowski K, Gaylord TG, Hardy RW (2007). Expanding the utilization of sustainable plant products in aquafeeds: a review. Aquac Res.

[19] Denison MS, Nagy SR (2003). Activation of the aryl hydrocarbon receptor by structurally diverse exogenous and endogenous chemicals. Annu Rev Pharmacol.

[20] Patisaul HB, Adewale HB (2009). Long-term effects of environmental endocrine disruptors on reproductive physiology and behavior. Front Behav Neurosci.

[21] Ortiz-Delgado JB, Scala E, Arellano JM, Úbeda-Manzanaro M, Sarasquete C. Toxicity of malathion at early life stages of the Senegalese sole, *Solea senegalensis* (Kaup, 1858): notochord and somatic disruptions. Histol Histopathol. 2017; 10.14670/HH-11-899. 10.14670/HH-11-899 28452045

[22] Sarasquete C, Úbeda-Manzanaro M, Ortiz-Delgado JB (2017). Effects of the soya isoflavone genistein in early life stages of the Senegalese sole, *Solea senegalensis*: thyroid, estrogenic and metabolic biomarkers. Gen Comp Endocrinol.

[23] Kim DJ, Seok SH, Baek MW, Lee HY, Na YR, Park SH (2009). Developmental toxicity and brain aromatase induction by high genistein concentrations in zebrafish embryos. Toxicol Mech Methods.

[24] DiMaggio MA, Kenter LW, Breton TS, Berlinsky DL (2016). Effects of dietary genistein administration on growth, survival and sex determination in southern flounder, *Paralichthys lethostigma*. Aquac Res.

[25] Akiyama T, Ishida J, Nakagawa S, Ogawara H, Watanabe SI, Itoh N, Shibuya M, Furaki Y (1987). Genistein, a specific inhibitor of tyrosine-specific protein kinases. J Biol Chem.

[26] Amakura Y, Tsutsmi T, Sasaki K, Nakamura M, Yoshida T, Maitani T (2008). Influence of food polyphenol on aryl hydrocarbon-receptor-signaling pathway estimated by in bioassay. Phytochemistry.

[27] Nagata S (1996). Fas-induced apoptosis, and diseases caused by its abnormality. Genes Cells.

[28] Wheatley SP, McNeish IA (2005). Survivin: a protein with dual roles in mitosis and apoptosis. Int Rev Cytol.

[29] Budke H, Orazi A, Neiman RS, Cattoretti G, John K, Barberis M (1994). Assessment of cell proliferation in paraffin sections of normal bone marrow by the monoclonal antibodies Ki-67 and PCNA. Mod Pathol.

[30] Kelman ZPCNA (1997). Structure, functions and interactions. Oncogene.

[31] Maga G, Hubscher U (2003). Proliferating cell nuclear antigen (PCNA): a dancer with many partners. J Cell Sci.

[32] Strzalka W, Ziemienowicz A (2011). Proliferating cell nuclear antigen (PCNA): a key factor in DNA replication and cell cycle regulation. Ann Bot.

[33] Ortego LS, Hawkins WE, Walker WW, Krol RM, Benson WH (1994). Detection of proliferating cell nuclear antigen in tissues of three small fish species. Biotech Histochem.

[34] Piñuela C, Rendón MC, González de Canales ML, Sarasquete C (2004). Development of the Senegal sole, *Solea senegalensis* forebrain. Eur J Histochem.

[35] Leung AY, Leung JC, Chan LY, Ma ES, Kwan TT, Lai KN, Meng A, Liang R (2005). Proliferating cell nuclear antigen (PCNA) as a proliferative marker during embryonic and adult zebrafish hematopoiesis. Histochem Cell Biol.

[36] Borucinska JD, Schmidt B, Tolisano J, Woodward D (2008). Molecular markers of cancer in cartilaginous fish: immunocytochemical study of PCNA, p-53, myc and ras expression in neoplastic and hyperplastic tissues from free ranging blue sharks, *Prionace glauca* (L.). J Fish Dis.

[37] Ortiz-Delgado JB, Fernández I, Sarasquete C, Gisbert E (2014). Normal and histopathological organization of the opercular bone and vertebrae in gilthead sea bream *Sparus aurata*. Aquat Biol.

[38] Bakke-McKellep AM, Penn MH, Salas PM, Refstie S, Sperstad S, Landsverk T (2007). Effects of dietary soyabean meal, inulin and oxytetracycline on intestinal microbiota and epithelial cell stress, apoptosis and proliferation in the teleost Atlantic salmon (*Salmo salar* L.). Br J Nutr.

[39] Sanden M, Olsvik PA (2009). Intestinal localization of PCNA protein and CYP1A mRNA in Atlantic salmon *Salmo salar* L. exposed to a model toxican. BMC Physiol.

[40] Dezfuli BS, Giari L, Lui A, Squerzanti S, Castaldelli G, Shinn AP (2012). Proliferative cell nuclear antigen (PCNA) expression in the intestine of *Salmo trutta trutta* naturally infected with an acanthocephalan. Parasit Vectors.

[41] Sirri R, Bianco C, De Vico G, Carella F, Bonaldo A, Sarli G (2014). Proliferation, apoptosis, and fractal dimension analysis for the quantification of intestinal trophism in sole (*Solea solea*) fed mussel meal diets. BMC Vet Res.

[42] Takle H, Andersen O (2007). Caspases and apoptosis in fish. J Fish Biol.

[43] Waring P, Müllbacher A (1999). Cell death induced by the Fas/Fas ligand pathway and its role in pathology. Immunol Cell Biol.

[44] Elmore S (2007). Apoptosis: a review of programmed cell death. Toxicol Pathol.

[45] AnvariFar H, Amirkolaie AK, Miandare HK, Ouraji H, Jalali MA, Üçüncü Sİ (2017). Apoptosis in fish: environmental factors and programmed cell death. Cell Tissue Res.

[46] Sakamaki K, Nozaki M, Kominami K, Satou Y (2007). The evolutionary conservation of the core components necessary for the extrinsic apoptotic signaling pathway, in Medaka fish. BMC Genomics.

[47] Ambrosini G, Adida C, Altieri DC. A novel anti-apoptosis gene, survivin, expressed in cancer and lymphoma. Nat Med. 1997;3:917–21. 10.1038/nm0897-917 9256286

[48] Ambrosini G, Adida C, Sirugo G, Altieri DC (1998). Induction of apoptosis and inhibition of cell proliferation by survivin gene targeting. J Biol Chem.

[49] Tamm I, Wang Y, Sausville E, Scudiero DA, Vigna N, Oltersdorf T, Reed JC (1998). IAP-family protein survivin inhibits caspase activity and apoptosis induced by Fas (CD95), Bax, caspases, and anticancer drugs. Cancer Res.

[50] Deveraux QL, Reed JCIAP (1999). Family proteins—suppressors of apoptosis. Genes Dev.

[51] Garg H, Suri P, Gupta JC, Talwar GP, Dubey S (2016). Survivin: a unique target for tumor therapy. Cancer Cell Int.

[52] Dinis MT, Ribeiro ML, Soares F, Sarasquete CA (1999). Review on the cultivation potential of *Solea senegalensis* in Spain and in Portugal. Aquaculture.

[53] Gisbert E, Ortiz-Delgado JB, Sarasquete C (2008). Nutritional cellular biomarkers in early life stages of fish. Histol Histopathol.

[54] Morais S, Aragão C, Cabrita E, Conceição LEC, Constenla M, Costas B (2014). New developments and biological insights into the farming of *Solea senegalensis* reinforcing its aquaculture potential. Rev Aquac.

[55] Darras VM, Houbrechts A-M, Van Herck SLJ (2015). Intracellular thyroid hormone metabolism as a local regulator of nuclear thyroid hormone receptor-mediated impact on vertebrate development. Biochim Biophys Acta.

[56] Úbeda-Manzanaro M, Ortiz-Delgado JB, Rebordinos L, Sarasquete C (2014). Expression profiling of the sex-related gene Dmrt1 in adults of the Lusitanian toadfish, *Halobatrachus didactylus* (Bloch and Schneider, 1801). Gene.

[57] Pfaffl MW (2001). A new mathematical model for relative quantification in real-time RT-PCR. Nucleic Acids Res.

[58] Sarasquete C, Gutiérrez M (2005). New tetrachromic VOF stain (type -III G.S.) for normal and pathological fish tissues. Eur J Histochem.

[59] Úbeda-Manzanaro M, Ortiz-Delgado JB, Sarasquete C (2016). Cloning and sequencing of Tert gene in gilthead seabream, *Sparus aurata*, and European seabass, *Dicentrarchus labrax*: expression patterns in germ and somatic cells. Agri Gene.

[60] Bernardos RL, Barthel LK, Meyers JR, Raymond PA (2007). Late-stage neuronal progenitors in the retina are radial Müller glia that function as retinal stem cells. J Neurosci.

[61] Ito Y, Tanaka H, Okamoto H, Ohshima T (2010). Characterization of neural stem cells and their progeny in the adult zebrafish optic tectum. Dev Biol.

[62] Schreiber M (2013). Flatfish: an asymmetric perspective on metamorphosis. Curr Top Dev Biol.

[63] Bejarano-Escobar R, Blasco M, Degrip WJ, Loyola-Velasco JA, Martín-Partido G, Morcillo JF (2010). Eye development and retinal differentiation in an altricial fish species, the Senegalese sole (*Solea senegalensis*). J Exp Zool.

[64] Olvisk PA, Torstensen BE, Berntssen MHG (2007). Effects of complete replacement of fish oil with plant oil on gastrointestinal cell death, proliferation and transcription of eight genes encoding proteins responding to cellular stress in Atlantic salmon *Salmo salar* L. J Fish Biol.

[65] Nabilsi NH, Broaddus RR, McCampbell AS, KH L, Lynch HT, Chen LM, Loose DS (2010). Sex hormone regulation of survivin gene expression. J Endocrinol.

[66] Dohi T, Beltrami E, Wall NR, Plescia J, Altieri DC (2004). Mitochondrial survivin inhibits apoptosis and promotes tumorigenesis. J Clin Invest.

[67] Luzio A, Matos M, Santos D, Fontaínhas-Fernandes AA, Monteiro SM, Coimbra AM (2016). Disruption of apoptosis pathways involved in zebrafish gonad differentiation by 17α-ethinylestradiol and fadrozole exposures. Aquat Toxicol.

[68] Chang HC, Doerge DR (2000). Dietary genistein inactivates rat thyroid peroxidase in vivo without an apparent hypothyroid effect. Toxicol Appl Pharmacol.

[69] Schmutzler C, Gotthardt I, Hofmann PJ, Radovic B, Kovacs G, Stemmler L (2007). Endocrine disruptors and the thyroid gland, a combined in vitro and in vivo analysis of potential new biomarkers. Environ Health Persp.

[70] Pelissero C, Le Menn F, Kaushick S (1991). Estrogenic effect of dietary soybean meal on vitellogenesis in cultured siberian sturgeon, *Acipenser baeri*. Gen Comp Endocrinol.

[71] Fernández I, Ortiz-Delgado JB, Darias MJ, Hontoria F, Andree KB (2017). Vitamin a affects flatfish development in a thyroid hormone signaling and metamorphic stage dependent manner. Front Physiol.

